# Results of Proficiency Testing for *Trichinella* in Poland, 2015–2019

**DOI:** 10.3390/jcm10225389

**Published:** 2021-11-18

**Authors:** Mirosław Różycki, Weronika Korpysa-Dzirba, Aneta Bełcik, Ewa Bilska-Zając, Jacek Karamon, Jacek Sroka, Jolanta Zdybel, Tomasz Cencek

**Affiliations:** Department of Parasitology and Invasive Diseases, National Veterinary Research Institute, Partyzantow Avenue 57, 24-100 Pulawy, Poland; weronika.korpysa@piwet.pulawy.pl (W.K.-D.); aneta.belcik@piwet.pulawy.pl (A.B.); ewa.bilska@piwet.pulawy.pl (E.B.-Z.); j.karamon@piwet.pulawy.pl (J.K.); jacek.sroka@piwet.pulawy.pl (J.S.); j.zdybel@piwet.pulawy.pl (J.Z.); tcencek@piwet.pulawy.pl (T.C.)

**Keywords:** *Trichinella spiralis*, proficiency testing, Poland

## Abstract

Trichinellosis is a zoonotic meat-borne disease caused by the nematodes of the genus *Trichinella*. Meat containing live *Trichinella* larvae is a source of infection. The examination of meat for *Trichinella* was introduced in 1869, but the digestion method for this did not appear in Poland until the late 1970s. Nowadays, the meat of all food animals susceptible to *Trichinella* spp. is examined in the frame of official *post mortem* control with the digestion method. The majority of laboratories in Poland meet the requirements of the ISO/IEC 17025 Standard (352 field laboratories). Laboratory personnel participate in quality control programs. This paper presents the results of proficiency tests (PTs) organized within 2015–2019 in Poland. Over this period, the laboratories examined 7580 samples (contamination levels: zero, one, three, and five larvae). Each laboratory was provided with a set of samples (one negative and three positive). Over 95% of the samples were considered correct in qualitative assessments, though the results of the quantitative evaluations were slightly lower, with 89% of samples being considered correct. Based on a sample evaluation, 88% of laboratories passed the PT comparison. A slight decrease was observed in the examination of samples spiked with five larvae, and great progress was achieved in samples containing three larvae. Low levels of sample contamination are sought after in laboratories but may make evaluations difficult. For this reason, we must consider increasing the number of larvae added to the samples in the next PTs.

## 1. Introduction

Trichinellosis is a zoonotic parasitic disease caused by nematodes of the *Trichinella* genus. Infection occurs after the ingestion of raw or undercooked meat containing live *Trichinella* larvae. The first cases of trichinellosis in Poland were described in 1865 in Greater Poland. Under the Prus partitions, pork meat intended for consumption has been tested for the presence of *Trichinella spiralis* since 1869, using the trichinoscopic method (TRM), which became obligatory under the Austria partition in 1883 [1]. In the Kingdom of Poland, and under Russian partition, meat inspection was the initiative of a veterinarians. After the First World War, Poland regained independence. The obligatory examination of pig meat for *T. spiralis* was introduced by the regulation of the President of the Republic of Poland in 1928. In 1953, the regulation extended the compulsory examination of pigs to an obligation to test the meat of wild boar and pigs slaughtered for owner consumption. Nowadays, over 18 million pigs, 120,000 wild boars, and 40,000 horses are examined for *Trichinella* spp. in Poland each year. In recent years, the number of condemned pig carcasses has usually reached no more than 10 per year. However, in 2013, over 68 pigs from one farm in East Poland were found to be *T. spiralis*-positive, and in the last decade, *T. spiralis* and *T. britovi* larvae were found in two horse carcasses (one in Poland and another in Italy) [2]. Currently, the main risk to humans is associated with the consumption of wild boar meat, due to the high prevalence of *Trichinella* spp. in these animals, reaching 0.5% [3,4].

In 2009, the pooled sample digestion method supported by a magnetic stirrer (MSD) was introduced to all abattoirs and wild game establishments according to EU regulation 2075/2005 [5,6]. In this method, the muscle tissues are dissolved in artificial stomach flu-id, and subsequently, larvae are concentrated and examined microscopically. This method is recommended by the EU Commission as a reference method and for routine use [7,8]. Digestion assays can be used on single or pooled muscle samples for isolation and identification [9]. Several variations of the digestion assay exist. The purpose of EU regulation no. 2075/2005 (replaced by EU regulation no. 2015/1375) was to implement the diagnostic methods in the same manner in all EU countries [10]. This task was given to European Reference Laboratory for Parasites (EURLP), designated to coordinate the work of National Reference Laboratories (NRLs) [5,6,7]. In 2005, in Poland, there were more than 1100 laboratories performing examinations of meat for *Trichinella* using TRM and MSD. In the years since, the number of laboratories has decreased, to a little above 600 in 2021, as a result of higher hygienic requirements, market consolidation, and rising criteria for official designation. To remove barriers in internal EU trade, analytic methods for official controls must be equivalent [11]. Thus, the management system, as well as the accreditation process and EU regulation 2015/1375, require laboratories to confirm their competence in proficiency testing (PT) [8,12]. At present, over 300 laboratories are certified for compliance with the ISO 17025 Standard. The list of designated by the Chief Veterinary Officer (CVO) and accredited laboratories is available at http://www.wetgiw.gov.pl (accessed on 18 November 2021). Interlaboratory comparisons are organized by NRL every year. The first round of PTs was organized by EURLP in 2006, but a well-established methodology was not introduced until 2013. In previous years (2006–2011), the methods for PT sample preparation were changing, as well as the reporting form or number of larvae in the sample [13,14]. Proficiency testing for *T. spiralis* detection brings a number of challenges. The most important of these are the easy preparation of the samples and the long survival of the larvae in the samples during transportation and laboratory preparation. Generally, at the beginning, all laboratories tried to use naturally infected meat samples or samples of meat previously tested with the trichinoscopic method to spike PT samples. Most laboratories in Europe initially used naturally or experimentally infected meat. Thanks to work presented by Vallee [15], partially digested larvae have been utilized for PTs. Since 2014, our own methodology with larvae protected by gelatin capsules has been used (Patent P.428256). This article describes the organization of PT for *T. spiralis* diagnostics in Poland between 2015 and 2019 and evaluates the quantitative and qualitative PT results.

## 2. Materials and Methods

### 2.1. Principles of Proficiency Testing Organization

Proficiency testing (PT) organized by the NRL for *Trichinellosis* is based on the guidelines of the ISO/IEC 17043 standard. The PT program has been accepted by the Polish Center for Accreditation (PCA). For organizational reasons, the NRL team is supported by regional coordinators (RC) appointed by the local veterinary officers (LVO). In 2014, the methodology of interlaboratory comparisons developed by the NRL was transferred to regional laboratories. Since 2015, interlaboratory comparisons at the regional level have been organized by regional coordinators. However, once every two years, field laboratories are required to participate in the PT program at the national level organized by the NRL. Following the plan approved by the Director of the National Veterinary Research Institute, regional coordinators submit a list of field laboratories designated to participate in the PT to the NRL. The number of laboratories participating in the tests in 2015–2019 varied from 365 to 394 (Table 1). Each laboratory was provided with a set of samples, individual codes, and passwords to access the PT website. Each set consisted of four samples: one negative, one on the detection limit (one larva), and two spiked with three and five larvae, respectively. The codes for laboratories and samples are unique and generated by a random number generator. After logging into the PT website, participants receive the methodology of the PT and information on how the results are evaluated. The task of the laboratory is to examine the received meat samples within a week. The online form for PT results submission has a unique mechanism preventing collusion. Sample positions for typing results are generated randomly and changed with each participant logging into the system. Just after the submission of the results, a partial report (qualitative evaluation) for an individual laboratory is automatically generated and can be printed or saved as a PDF file. These reports are supposed to be submitted to the LVO and RC within a week after the PT. Comprehensive final reports are prepared within a month after the PT rounds and are sent to the CVO, LVO, and RC. Each report contains information on the participant’s test results, an interpretation of the results, and discussion, along with a comparison with the previous test round. Additionally, the quality system in the entire region is assessed based on the results of the tests of samples contaminated with one larva. The LVO together with the RC imposes corrective actions to improve the quality of the examination. Proposed corrective actions are brought up in consultations with the NRL. After their completion, the NRL prepares the samples for laboratory re-testing. The re-testing reports are sent to the CVO, LVO, and RC.

### 2.2. Sample Preparation

Before sample preparation, the lists of laboratories and labels were printed. Each sample was labeled with a laboratory code and an individual sample number. Each set (four samples) includes printed information about the website, a user ID, and a password. Each batch of PT samples was stored/transported under controlled conditions at 2–6 °C. The stability of the samples was evaluated in an earlier study (unpublished data). Each shipment was accompanied by a termoregistrator (LB-516T, LAB EL) recording the journey’s start and end temperatures with one-minute intervals, which allowed us to trace the conditions of transport. The transport took place in LVO vehicles, ensuring proper storage conditions, a fast delivery of samples, and safety. Samples were prepared as follows: The fresh meat was purchased at a butcher’s shop. Fresh pork ham was minced twice and divided into samples of 50 g each, and gelatin capsules with a defined number of *T. spiralis* larvae were added to the middle of the sample. The genus of larvae was confirmed by PCR at NRL. Placing the capsule in the center of the sample limits the access of oxygen, allowing adequate humidity to be maintained. Samples had three different *T. spiralis* contamination levels (zero, one, three, and five larvae). Each set sent to the laboratory also contained a blank sample with gelatin capsules without larvae. Samples containing single larva were used to assess the quality of the management system in each province.

### 2.3. Capsules’ Preparation

As a source of *T. spiralis* larvae, naturally infected wild boar meat was used. Portions of wild boar meat weighing 100 g were dissolved in the artificial digestion fluid with a standard MSD (2 L of water preheated to 48 °C, 16 mL of 25% hydrochloric acid, and 10 g of pepsin 2000 FIP). The meat was chopped with a blender (MSM 2650B, Bosch, Gerlingen Germany) with the addition of a small amount of digestion fluid and transferred to a 3 L glass beaker with the remaining digestion fluid. The glass beaker was placed in a magnetic stirrer (MR Hei-Tec, Heidolph, Schwabach, Germany) and incubated at 45 ± 1 °C/400–500 rpm/45 min. The digestion fluid was filtered through a 180 µm sieve directly into a 3000 mL conical glass. After two consecutive sedimentations and washing, 10 mL of the final sediment was transferred into a Petri dish. The single larva was transferred into a solid base made of 8%, 300 Bloom type A pig-skin gelatin (Sigma-Aldrich, Albuch, Germany) and 0.9% natrium chloride (POCH, Gliwice, Poland). A gelatin medium was prepared the day before the experiment. This medium was divided into two parts. One part was poured into a Petri dish (0.7 mm thickness). After the medium solidified, small wells were carefully cut out with a corkbore. A thin layer of gelatin medium was applied to the bottom of the wells (max. one-third of the well height). After the bottom layer had solidified, the medium was ready for the transfer of the larvae. At the same time, the second part of the medium was liquefied and cooled to a temperature of 46–47 °C (higher temperatures may destroy the larvae). The larvae from the sediment were counted under a microscope (40× magnification) and transferred with a transparent pipette to the bottom of the wells. The larvae were carefully covered (to prevent foaming) with a second gelatin layer. The solidified medium was completely transparent and the presence of the larvae was re-confirmed under the microscope by another technician. The required number of larvae was taken from a solid medium using a wider corkbore. Each of the gelatin capsules was checked twice at each stage of preparation before placing them into the meat samples, ensuring that the number of *T. spiralis* larvae used to contaminate the meat samples was strictly determined. The *Trichinella* genus was examined with PCR according to the EURLP guidelines [16].

### 2.4. Laboratory Testing

All laboratories in Poland for official control measures use only the reference method described in (EU) Commission Implementing Regulation 2015/1375, so laboratories that participated in the PT performed only the MSD. Samples delivered to the laboratory did not require any additional processing. From that moment, laboratories were obligated to finish the examination within five working days. Forms describing the condition of the sample upon arrival to the laboratory were available for PT participants on the website. It was recommended wherever possible, that different technicians should perform the examination of individual samples. Accordingly, when reporting the results, the name of the person who performed the test should have been provided for each sample.

### 2.5. Interpretation of the PT Results

#### 2.5.1. Qualitative Assessment

The qualitative result of the examination of the sample was considered as follows:

“correct”—if *T. spiralis* larvae were found in contaminated samples and if larvae were not found in the blank sample.

“incorrect”—if *T. spiralis* larvae were not detected in contaminated samples or if larvae were detected in the blank sample.

Laboratories were evaluated based on the results of the examination of samples at levels of zero, three, and five; one unsatisfactory result leads to a negative assessment of the laboratory.

#### 2.5.2. Quantitative Assessment

Following the guidelines given by EURLP, the absolute value |Δ| of the difference between the laboratory result and the reference value was used for the quantitative assessment of the PT results.

Depending on the value of the indicator |Δ|, the quantitative results were considered as: |Δ| ≤ 2—“satisfactory”, |Δ| = 3—“doubtful”, and |Δ| > 3—“unsatisfactory”. For samples spiked with three larvae, |Δ| ≤ 2 is satisfactory, without a doubtful evaluation.

If laboratories found two larvae during examination of samples at level five, these findings were considered doubtful. Furthermore, the quality of the system was evaluated for each province based on the results of samples analyzed at the level of the detection limit of the MSD (one larva). When assessing the test system, the assumptions for the validation of the method given by Forbes, indicating the correctness of the research, were that 75% of laboratories within the region should detect *T. spiralis* larvae in samples at the detection limit [6,17].

## 3. Results

### 3.1. Qualitative Assessment of Laboratory Performance

Over 2015–2019, 1895 laboratories participated in the organized PTs. In 2015, 394 laboratories were involved, 374 in 2016, 384 in 2017, 347 in 2018, and 378 in 2019. The qualitative results of the sample examination are presented in Table 1.

Depending on the level of contamination, the percentage of positively assessed samples in the qualitative assessment varied from 89% to 98%. The PT results within the level are shown in Table 2.

**Figure 1:**
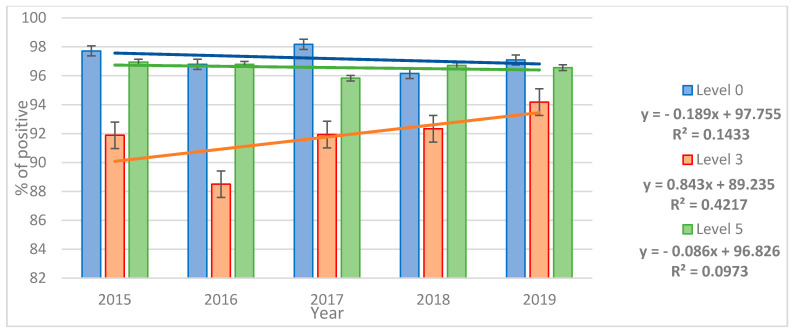
The percentage of correct results in particular years and the formation of a trend.

The qualitative performance of laboratories and trend analyses are shown in Figure 1.

The real progress in terms of the qualitative assessment can be observed in the examination of samples spiked with three larvae. No significant changes were observed in the examination of *T. spiralis*-free samples or samples spiked with five larvae.

In the laboratory evaluation, the laboratory was assessed negatively if it reported at least one incorrect result on the levels of zero, three, or five larvae. The results of the qualitative evaluation of laboratory performance are presented in Table 3.

Very slight progress can be observed in the qualitative performance of laboratories year to year (trend line described by the equation: y = 0.2953x + 86.797). However, a fluctuation of the results can be observed in the year-to-year comparison. The lowest percentage of laboratories that passed the PT comparison was observed in 2016 (85%), while the highest was observed in 2019, reaching 89%.

### 3.2. Quantitative Assessment of Laboratory Performance

The final report includes qualitative and quantitative laboratory assessments. The quantitative assessment was based on the results for the absolute Delta value (|Δ|), which was calculated as the difference between the laboratory results and the reference value (|Δ| = X lab − X ref).

#### 3.2.1. Sample Quantitative Evaluation

The results of the samples quantitative evaluation are presented in Table 4.

**Figure 2:**
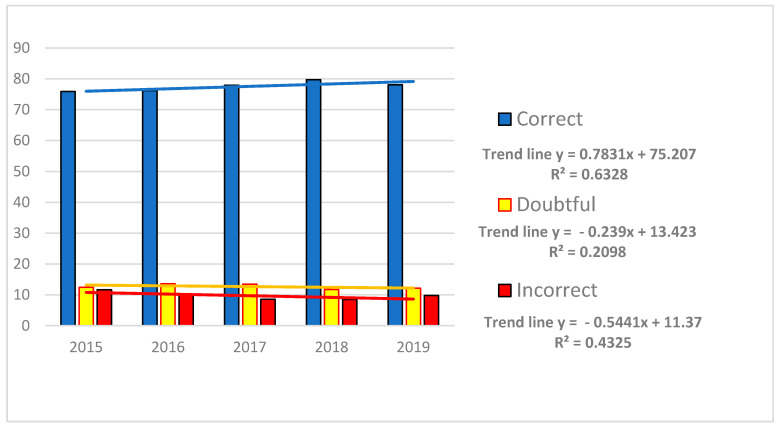
The percentage of samples assessed as correct, doubtful, and incorrect in quantitative assessment.

A downward trend in terms of the percentage of negative and doubtful results, in favor of positive results, can be observed in Figure 2.

The quantitative assessment of the PT test results, based on the criteria described previously, showed that most laboratories reported single non-compliant results as opposed to more than one. 

#### 3.2.2. Sample Quantitative Evaluation

Based on the reported results, the laboratories were evaluated. The laboratory performance is shown in Table 5.

The obtained results are presented in Table 6 as the percentage of laboratories reporting non-conforming results for one or more samples.

Overall, the percentage of samples assessed as correct ranged from 94% to 96%, but the percentage of laboratories that obtained “consistent” results in the qualitative assessment ranged from 85% to 89%. The majority of these laboratories (203) obtained a “non-consistent” result in a single sample, accounting for 14% of all the laboratories participating in the study.

### 3.3. Assessing the Quality Performance with a Sample Contaminated with One Larva

The single larva contamination study did not apply to the single-laboratory evaluation but served as an indicator of the system performance within the region. The evaluation was based on the percentage of true positive results at the level of one larva. It was estimated that ≥75% of the results were correct at this level, proving quality system efficiency. Less than 75% of positive results should be treated as a signal for preventive actions such as training, equipment checks, or interlaboratory tests. In Table 7, the results with a year-to-year comparison are presented. 

The best results were obtained in 2017, reaching 76% of all samples. On the contrary to rising trends in PTs, the obtained results are not satisfactory. The overall tendency is to decrease and the trend line can be described as y = −0.8x + 73, R² = 0.1013. The presented research results generally describe the tendency of the research quality system in the whole country. Yet, there are regional differences reflecting the examination capacity and quality systems. The significant differences between regions over the years disappeared and the positive reported results stabilized at a level close to 70%.

## 4. Discussion

PTs have been organized by the Polish NRL since 2005. However, their methodology has changed in the subsequent years. Over this time, the introduction of the MSD method has been an important factor influencing the methodology of conducting PT research. This methodology was finally established in 2014–2015 and has been used with slight changes to the present day. In this study, the results of the qualitative assessment indicate that the best results were obtained by the laboratories in the analysis of the samples contaminated with five larvae. Over 96% of the laboratories obtained “consistent” results in subsequent years. When considering the level of contamination with three larvae, the percentage of “consistent” results ranged from 89% to 93%, which is understandable given the greater difficulty of testing such samples. Correct results for blank samples ranged between 96% and 98% in different years.

Trackback studies on PTs indicate that the level of reported correct results at all levels of contamination since 2014 bears a slightly increasing trend. Numerous consultations and the transition of knowledge by the ICT and EURLP have significantly sped up the implementation of quality assurance in the PT methodology [10,18,19]. Guidelines delivered at meetings organized by the ICT and EURLP have been transposed into Polish legislation as the CVO Guidelines. Proficiency comparisons for *T. spiralis* have evolved over the years, from a simple training tool to a test quality control. Real progress in the quality of examination can thus be observed from 2009 onwards (unpublished data). In the years 2009–2014, proficiency tests were characterized by a significant increase in the quality of the tests. This trend continued in 2015–2019 but flattened significantly.

The percentage of consistent results in qualitative evaluations in previous years (2009–2014) was high, ranging from 92% to almost 95%, but it should be noted that in previous years, these studies were carried out on samples contaminated with up to three times the load of larvae [20]. A higher amount of added larvae significantly increases the probability of finding one larva [21]. The results obtained in 2015–2019 show that the quality of the tests increased with a reduction in the number of larvae in the sample, which prompted laboratories to conduct more precise work. Since artificial digestion lacks internal controls, proficiency testing must be carried out at regular intervals [8]. The obtained results are comparable with those presented by German NRL, reporting 94% correctly assessed samples and 80% of the laboratories. The differences between the two countries’ results may be due to the different levels of contamination, as in Germany, they use higher contamination levels than in Poland (3, 4, 8, and 15 larvae) [7]. Slightly better results were reported by Marucci et al. on PT carried out by the EURL for NRLs in 2007–2015. The percentages of NRLs that obtained “consistent” results in the analyzed years were 83%, 92%, 89%, 86%, 86%, 100%, 87%, 100%, and 88%, respectively [5]. Lower numbers of larvae spiked in the sample decreased the relative differences among the results reported by the NRLs [5].

In the past five years, the quality of examinations has slightly increased year to year; however, certain warning signals appeared in 2018 (one larvae examination). In 2017, laboratories in a few regions had their accreditation suspended, in one case leaving just one accredited laboratory within a region (Zachodniopomorskie, Śląskie), as a result of (EU) regulation 2017/625 coming into force. This regulation indicates accreditation as the instrument of choice to ensure high performance by official laboratories, but enables laboratories under certain conditions to reduce their costs by replacing the accreditation system with another equivalent quality system. This is true in cases where laboratories independently prepare procedures and quality systems. Poland is an example of a slightly different approach to the problem of accreditation of field laboratories. The integration of these laboratories into the existing quality system at the regional veterinary laboratories omitted that problem. This approach has allowed for a significant reduction in costs and has accelerated the accreditation process of laboratories. In response to this trend and the related risk of lowering the quality of examination, regional laboratories have been trained and obliged to conduct interlaboratory comparisons as a quality control tool in both accredited and non-accredited laboratories. Since this is a lengthy process and one that has additionally been hampered by the COVID-19 pandemic, the results of these activities can be assessed in the future.

## 5. Conclusions

The decreasing number of *T. spiralis* infections in domestic pigs in developed countries has led to a significant decline in human trichinellosis; however, it remains a potential risk due to the presence of the parasites in wildlife. The diagnosis and control of *T. spiralis* infection in food animals are fundamental to ensuring consumer protection from this parasite. In this context, the effectiveness of the meat inspection system highly depends on the application of proper quality assurance standards. The use of sample spiked with low numbers of larvae prompts laboratories to work better and allows for abnormalities to be detected and corrected at the laboratory level. On the other hand, the use of samples with a low level of contamination increases the risk of incorrect laboratory evaluation since the distribution of results reported by laboratories is non-normal. The solution to this problem would be to use samples contaminated with other parasites or artefacts. The final goal of maintaining the quality by examining all laboratories is to decrease the threat posed by *T. spiralis* to consumers. The results of the PT tests carried out indicate that the quality of the tests performed in Polish veterinary laboratories does not differ from the results obtained in European laboratories; however, the results indicate a possible need for further actions. Yet, the developed methodology significantly increases the precision by adding a specific number of parasites to meat samples. It also ensures the long survival of *T. spiralis* in samples, which eliminates errors related to the toxic effect of oxygen, transport, and storage conditions.

## Figures and Tables

**Table 1:** Sample qualitative results of PT from 2015 to 2019 (all levels: zero, three, and five larvae).

Year	Number of Laboratories Participating in the Study	Total Number of Sent Samples (Contamination Level: Zero, Three, Five)	Total Number of Sample Correctly Assessed	Total Number of Sample Incorrectly Assessed	% of Sample Correctly Assessed
2015	394	1182	1129	53	96
2016	374	1122	1055	67	94
2017	384	1152	1098	54	95
2018	365	1095	1041	54	95
2019	378	1134	1088	46	96
Total	1895	5685	5411	274	95

**Table 2:** Sample qualitative evaluation by the level of contamination in PT from 2015 to 2019.

Number of Larvae per Sample	Year/Sample									
	2015/394		2016/374		2017/385		2018/365		2019/378	
	C ^1^/I ^2^	% C	C/I	% C	C/I	% C	C/I	% C	C/I	% C
0	385/9	98	362/12	97	378/7	98	351/14	96	367/11	97
3	362/32	92	331/43	89	354/31	92	337/28	92	356/22	94
5	382/12	97	362/12	97	369/16	96	353/12	97	365/13	97

^1^ C, correct; ^2^ I, incorrect.

**Table 3:** Laboratory qualitative results of laboratory evaluation.

Year	Number of Laboratories Participating in the Study	Total Number of Laboratory with Correct Results	Total Number of Laboratory Reporting Incorrect Results	% of Laboratories Passed PT Comparison
2015	394	349	45	89
2016	374	318	56	85
2017	385	343	42	89
2018	365	315	50	86
2019	378	338	40	89

**Table 4:** Sample quantitative assessment.

Year	Number of Samples in Quantitative Assessment					
	Number of Samples per Level	Level 0	Level 3	Level 5	Level 5	Level 5
		|Δ| = 0 Correct	|Δ| ≤ 2 Correct	|Δ| ≤ 2 Correct	|Δ| = 3 Doubtful	|Δ| > 3 Incorrect
2015	394	385	362	299	49	46
2016	374	362	331	285	51	38
2017	385	378	354	300	52	33
2018	365	351	337	291	43	31
2019	378	367	356	295	46	37
**% of Samples in Quantitative Assessment**						
2015	394	98	92	76	13	12
2016	374	97	89	76	14	10
2017	385	98	92	78	14	9
2018	365	96	92	80	12	8
2019	378	97	94	78	12	10

**Table 5:** Laboratory quantitative evaluation by the level of contamination in PT from 2015 to 2019.

Year	Number of Laboratories Participating in the Study	Laboratories Quantitative Evaluation	
		Number and % Negatively Evaluated Laboratories ^1^	Number and % of Positively Evaluated Laboratories ^2^
2015	394	70/18	324/82
2016	374	77/21	297/79
2017	385	54/14	331/86
2018	365	65/18	300/82
2019	378	60/16	318/84

^1^ Laboratories reporting doubtful results twice or more were evaluated negatively. ^2^ Laboratories reporting doubtful results once were evaluated positively.

**Table 6:** Laboratories reporting one or more incorrect results.

Year	Total Number of PT Participants	Number of Laboratories Reporting One Negative Result		Number of Laboratories Reporting Two Negative Results		Number of Laboratories Reporting Three Negative Results	
2015	394	55	14%	14	4%	1	0.3%
2016	374	64	17%	12	3%	1	0.3%
2017	385	33	9%	20	5%	1	0.3%
2018	365	57	16%	8	2%	0	0.0%
2019	378	48	13%	12	3%	0	0.0%

**Table 7:** Results of examinations of samples spiked with one larva.

Year	Number of Laboratories Participating in the Study	Level One Larva Incorrect Results	% Positive Findings
2015	394	106	73
2016	374	113	68
2017	385	92	76
2018	365	123	66
2019	378	113	70
